# Internal Colonization of *Salmonella enterica* Serovar Typhimurium in Tomato Plants

**DOI:** 10.1371/journal.pone.0027340

**Published:** 2011-11-09

**Authors:** Ganyu Gu, Jiahuai Hu, Juan M. Cevallos-Cevallos, Susanna M. Richardson, Jerry A. Bartz, Ariena H. C. van Bruggen

**Affiliations:** 1 Emerging Pathogens Institute and Department of Plant Pathology, University of Florida, Gainesville, Florida, United States of America; 2 Department of Plant Pathology, University of Florida, Gainesville, Florida, United States of America; Institute for Genome Sciences, University of Maryland School of Medicine, United States of America

## Abstract

Several *Salmonella enterica* outbreaks have been traced back to contaminated tomatoes. In this study, the internalization of *S. enterica* Typhimurium via tomato leaves was investigated as affected by surfactants and bacterial rdar morphotype, which was reported to be important for the environmental persistence and attachment of *Salmonella* to plants. Surfactants, especially Silwet L-77, promoted ingress and survival of *S. enterica* Typhimurium in tomato leaves. In each of two experiments, 84 tomato plants were inoculated two to four times before fruiting with GFP-labeled *S. enterica* Typhimurium strain MAE110 (with rdar morphotype) or MAE119 (without rdar). For each inoculation, single leaflets were dipped in 10^9^ CFU/ml *Salmonella* suspension with Silwet L-77. Inoculated and adjacent leaflets were tested for *Salmonella* survival for 3 weeks after each inoculation. The surface and pulp of ripe fruits produced on these plants were also examined for *Salmonella.* Populations of both *Salmonella* strains in inoculated leaflets decreased during 2 weeks after inoculation but remained unchanged (at about 10^4^ CFU/g) in week 3. Populations of MAE110 were significantly higher (P<0.05) than those of MAE119 from day 3 after inoculation. In the first year, nine fruits collected from one of the 42 MAE119 inoculated plants were positive for *S. enterica* Typhimurium. In the second year, *Salmonella* was detected in adjacent non-inoculated leaves of eight tomato plants (five inoculated with strain MAE110). The pulp of 12 fruits from two plants inoculated with MAE110 was *Salmonella* positive (about 10^6^ CFU/g). Internalization was confirmed by fluorescence and confocal laser microscopy. For the first time, convincing evidence is presented that *S. enterica* can move inside tomato plants grown in natural field soil and colonize fruits at high levels without inducing any symptoms, except for a slight reduction in plant growth.

## Introduction

Fruits and vegetables, in particular leafy greens and fruit that are consumed raw, are increasingly recognized as vehicles for transmission of human enteric pathogens. Despite the increased importance of fresh produce as a source of enteric pathogens for humans, there is currently limited knowledge about contamination points in the supply chain or about the mechanism by which human pathogens colonize and survive on or in fruits and vegetables [1].

*Salmonella enterica* is the most frequently encountered pathogen associated with foodborne illness in the United States [2], [3]. Consumption of contaminated produce has been implicated in many of the salmonellosis outbreaks in recent years [4]. In particular, *Salmonella-*contaminated tomatoes have led to several multistate and international outbreaks, each involving hundreds of cases [5], [6], [7], [8], [9]. Contamination of produce may occur in the processing stage but sources of contamination have also been associated with certain production fields [10], [11], [12]. However, little is known about the routes of contamination and potential internalization in plants [13].

During crop production, irrigation water, particularly if applied overhead, could be an important source of contamination of plants with *Salmonella* [14], [15]. Foliar applications of fertilizers or pesticides where contaminated water was used to dilute the formulated products could also contaminate plants. Many pesticide formulations include surfactants, which enable the spray suspension to spread more uniformly over waxy plant surfaces. Surfactants differ chemically and in their abilities to reduce the surface tension of water and penetrate into plant surfaces. Surfactants that enhance penetration of aqueous solutions into plant surfaces, like trisiloxanes, are commonly used in herbicide formulations [16]. Silwet L-77, an organo-silicone surfactant based on trisiloxane ethoxylate, is considered a “super spreader” due to its effect on the water/cuticle interface. This surfactant is a component for many agro-chemical products on the market, including herbicides, insecticides, fungicides, plant growth regulators, fertilizers and micronutrients, at a concentration of 0.025% to 0.1% [17]. Some trisiloxane surfactants were shown to enhance the dispersal of foliar bacterial diseases to a greater extent in a simulated citrus nursery than did several other spreader/ stickers [18]. In contrast, Tween 20™ (polyoxyethylene sorbitan monolaurate) is a non-ionic surfactant that is widely used in agricultural applications, but appears to be just a spreader. It does not appear to enhance penetration of plant surfaces by aqueous solutions [19]. The effects of surfactants such as the trisiloxane products on the risk of contamination of crops plants with *S. enterica* have been insufficiently documented thus far.

*S. enterica* can colonize seeds [20], [21], sprouted seeds [22], leaves [23], [24], [25], [26] and fruit [27], [28] of a variety of plant species. The interaction with plants can depend on the particular serovar [23]. For example, while *S. enterica* Typhimurium, Enteritidis and Senftenberg adhered efficiently to leafy vegetables, others (Arizona, Heidelberg and Agona) did not [29].

The surface morphology of different strains of *Salmonella* appears to affect their survival and multiplication on plants. Pilus curli (also known as fimbriae), encoded by *agfB*, seemed to play an important role in adhesion of serovars Enteridis and Newport to alfalfa sprouts [30]. Curli and cellulose can also play a role in attachment of serovar Typhimurium to parsley [31]. Besides curli, the O antigen capsule (encoded by *yihO*) and cellulose synthesis (encoded by *bcsA*) have been implicated in adhesion of serovar Enteritidis to alfalfa sprouts [32]. Curli, cellulose and capsules are all regulated by *agfD* which contributes to the formation of bacterial rdar morphotype, which forms distinct, rough and dry colonies [33]. The wild type *Salmonella* rdar morphotype is not phenotypically stable and is highly dependent on environmental conditions, like lower temperature (<30°C), nutrient limitation or low osmolarity [34], [35], [36]. Previous researchers concluded that the rdar morphotype was important for environmental persistence with increased resistance to desiccation and antimicrobial agents [33], [37], [38], [39], [40].

In comparison to bacterial attachment to plant surfaces, the internal movement and translocation of *Salmonella* in plants have not been investigated in detail. Inoculation of tomato plants with *Salmonella* by dipping whole seedlings in a suspension with a cocktail of strains resulted in surface contamination of fruits on plants irrigated by automated drip tubes [41], but systemic translocation in the plants was not investigated. Several serovars of *Salmonella* were able to invade root tissues and spread into shoots [42], [43], but again, systemic translocation was not demonstrated.

In this study, we investigated the internalization of *S. enterica* Typhimurium into tomato plants via leaves, and evaluated the effects of surfactants and the bacterial rdar morphotype on internalization.

## Methods

### Bacterial strains and plant preparation

*S. enterica* Typhimurium strains MAE110 (*PagfD*1, rdar: aggregate/multicellular phenotype) and MAE119 (Δ*agfD*101, saw: smooth colony morphology) were kindly provided by Dr. Ute Romling [44], [45]. These strains carry kanamycin resistance and green fluorescent protein (GFP) genes on the chromosome and were derived from MAE52 and MAE51, respectively, after transformation with the PAG408 mini-transposon [46]. Different from their wild type strain *S. enterica* Typhimurium ATCC 14208, MAE110 constantly presents the rdar morphotype, whereas MAE119 has completely lost the rdar morphotype and develops shiny and smooth colonies [36], [44], [45], [47]. Bacterial cultures were stored in Luria-Bertani (LB) broth containing 25% glycerol at −80°C. For each experiment, a loopful of the stored culture was added to shake cultures (150 rpm) of LB broth (50 µg/ml kanamycin), grown for 18 to 20 h at 37°C. The cultures were harvested by centrifugation. The pellets were suspended in sterile distilled water (SDW) to an optical density of 0.46, which approximates 10^9^ CFU/ml.

Tomato seeds (*Solanum lycopersicum* ‘Florida Lanai’, a small-fruited compact variety, not commercially available) were kindly provided by Dr. Jane Polston at the University of Florida. These seeds were surface disinfected with 1 M HCl for 30 min. Seeds were germinated in potting mix. At 2 weeks post-seeding, seedlings were transplanted to sandy loam soil in 15-cm diameter pots placed on a saucer to collect runoff water. Sandy loam soils were collected from the Plant Science Experiment Station of the University of Florida at Citra, Florida, with typical fertilizer, fungicide, insecticide and herbicide application schedules (conventional soil) and from a certified organic farm where vegetables were grown organically in the past five years (organic soil). The soil organic matter content was 1.33% in the conventional soil and 2.33% in the organic soil and the pH was 6.5 in both soils. In this paper, the results obtained for both soils are lumped. Water was applied to the pots at a 2-day interval and fertilization was applied every 2 weeks as 150 ml half-strength Hoagland solution (pH 6.8). Plants were grown in a biological safety level 2 greenhouse equipped with ridge vents, a cooling air conditioning unit and a gas heater. For the first experiment red and blue LED lights (LGL Technologies, Inc., Barnesville, MD) were used with a 14/10 day/night cycle. For the second experiment, plants were exposed to natural light only. The temperature fluctuated between 23°C and 33°C, with an average temperature of 28°C.

### Inoculation of tomato leaves with *S. enterica* Typhimurium with or without surfactants

The effect of surfactants on penetration and colonization of leaves by *Salmonella* was examined prior to tests on *Salmonella* internalization and possible translocation in tomato plants. Tween 20™ and Silwet L-77 (Sigma Chemical Co., St. Louis, MO) were added to suspensions of *Salmonella* prior to leaflet dip inoculation. Eighteen 8-week-old tomato plants were inoculated with a *S. enterica* MAE110 (10^9^ CFU/ml) suspension containing 0.025% (v/v) Tween 20 ™, Silwet L-77 or SDW and placed on a greenhouse bench in a completely randomized design. For inoculation, three leaflets on each of two branches per plant were dipped into one of the three *Salmonella* suspensions for 30 s. At 7 and 14 days post inoculation, inoculated leaflets were immersed in 70% alcohol for 20 s and then 0.6% sodium hypochlorite for 10 s and rinsed 3 times by SDW to eliminate surface populations of bacteria. One 12-mm leaf disc was taken with a sterile cork borer from each leaflet and ground in 1 ml SDW and plated on LB plates (50 µg/ml kanamycin) after preparing a ten-fold dilution series. Samples (100 µl) of appropriate dilutions were spread onto LB agar plates containing 50 µg/ml kanamycin. The Petri plates were incubated at 37°C overnight. Numbers of *S. enterica* Typhimurium colonies on each Petri plate were determined by counting green fluorescent CFU's using a UV lamp (UVGL-25, Entela Inc., USA). All plates were checked under UV light to exclude the possibility of counting colonies that were not the gfp-marked *Salmonella* strains. Very few unidentified bacterial colonies were found on the LB agar with kanamycin; these did not show green fluorescence under UV light.

### Inoculation of tomato leaves with *S. enterica* Typhimurium for the internalization experiments

*Salmonella* internalization experiments were conducted twice in 2 years using a randomized complete block design. In each experiment, 126 tomato plants were evenly divided over seven blocks located on three greenhouse benches. Eighteen plants in each block were randomly inoculated with GFP labeled *S. enterica* Typhimurium strain MAE110, MAE119 or with SDW as control (six plants per treatment per block). Inoculation was carried out by dipping three leaflets on each of two branches per plant into 10^9^ CFU/ml *Salmonella* suspension with 0.025% (v/v) Silwet L-77 for 30 s. Control plants were inoculated with the same amount of SDW with 0.025% (v/v) Silwet L-77. Tomato plants were inoculated in weeks 5 and 10 after planting seeds in year 1, and in weeks 5, 8, 9 and 10 in year 2.

### Leaf sampling and testing procedure

In year 1, inoculated tomato leaflets were sampled 7 days after inoculation. In year 2, inoculated leaflets and non-inoculated adjacent leaflets were sampled 3 h, 1, 3, 5, 7, 14, 21 days after each inoculation. At each sampling time, two inoculated leaflets and one non-inoculated adjacent leaflet were removed from three randomly selected plants of each treatment in each block. Two 12-mm leaf discs were taken with a sterile cork borer from each inoculated leaflet. One of the two leaf discs was treated to eliminate surface populations of bacteria by dipping the disc in 70% alcohol for 20 s and then in 0.6% sodium hypochlorite for 10 s. Thereafter, leaf discs were rinsed 3 times by SDW. Both of the two discs with or without the surface treatment were ground in 1 ml SDW, the extract was diluted 10-fold in phosphate buffered saline (PBS) and 0.1 ml aliquots of the appropriate dilutions were spread over LB plates (50 µg/ml kanamycin) after preparing a 10-fold dilution series. Adjacent non-inoculated leaves were ground and enriched in LB broth (50 µg/ml kanamycin) overnight at 37°C. The number of *Salmonella* colonies was counted as described above.

### Fluorescence and confocal laser microscopy

In year 2, three inoculated leaflets were sampled 1 day after the second inoculation from each of the eight plants inoculated with *Salmonella* and eight control plants. Five days later, three non-inoculated adjacent leaves and one adjacent stem from each of the eight plants were also collected for fluorescent microscopic analysis as described previously [42]. In brief, the plant tissues were fixed overnight in 10% Neutral Buffered Formalin (Fisher Scientific Company, Middletown, VA) and then washed in PBS (pH 7.4) and soaked in 20% sucrose solutions (w/v) in PBS overnight at 4°C. Next, the samples were embedded in Tissue-Tek OCT compound (Miles, Elkhart, IN). About 40 tissue sections of 15 µm or 30 µm thickness were cut horizontally or vertically from each sample with a cryostat (Microm HM 500 O; Microm Laborgerate GmbH, Waldorf, Germany) at −20°C. The samples were transferred to slides and mounted in anti-fade mounting medium (Vector Laboratories).

GFP-labeled *Salmonella* cells in the tissue sections were observed with a fluorescence microscope (Leika DM4000 B; Leika, German) and a confocal laser scanning microscope (Olympus IX81-DSU; Olympus, Japan). The tissue sections were scanned for fluorescent bacteria under light with an excitation wavelength of 488 nm and a BA505-525 emission filter (GFP). Use of an excitation/emission wavelength of 541/572 nm (TRITC) enabled distinction of *Salmonella* cells from chlorophyll and vascular tissue auto-fluorescence under the GFP filter. Time lapse microscopy of a single field was employed.

### Fruit sampling and testing procedure

Ripe (fully red in color) tomatoes were picked by hand, placed in a plastic zip-lock bag and transported to the lab. Each tomato was placed in a sterile plastic bag with 30 ml of 0.1% sterile peptone water. Potential surface populations were dislodged by sonicating the bags for 5 min in an ultrasonic cleaner (Bransonic 5200, Branson Ultrasonics Corp., Danbury, CT). The *Salmonella* population in the peptone wash suspension was enriched and enumerated as described above. The fruit samples were then immersed in 70% alcohol for 2 min and then rinsed twice in SDW. Each fruit was vertically cut into halves with a sterile knife. Tomato halves were placed directly with cut-side-down for 1 min on LB agar plates supplemented with 50 µg/ml kanamycin. The halves then were removed from LB plates. The plates were incubated at 37°C overnight. *S. enterica* Typhimurium colonies on each Petri plate were determined by counting green fluorescent CFUs using a UV lamp. The pulp of *Salmonella* contaminated tomato fruits in ziplock bags was crushed by hand to form a pulp slurry, and then transferred into a 50-ml centrifuge tube and vortexed for 3 min. Thereafter, 1 ml of the slurry was used to establish tenfold dilution series with 0.1% peptone water. Aliquots (100 µl) of appropriate dilutions were spread onto LB agar plates containing 50 µg/ml kanamycin. The plates were incubated at 37°C overnight. Numbers of *Salmonella* colonies were counted as described above.

### Injection of *S. enterica* Typhimurium into peduncles

54 pink fruits with about 0.5 cm long peduncles were picked by hand from non-inoculated healthy plants. The weight of these individual tomatoes ranged from 27 to 44 g. Suspensions of *S. enterica* Typhimurium strains MAE110 and MAE119 were prepared separately as described above. Ten µl inoculum suspensions with a density of 10^4^ CFU/ml were injected into peduncles about 0.4 cm deep with the aid of sterile syringe needles (0.46 mm O.D., 13 mm Length). The opening caused by the needle was sealed with molten paraffin immediately after inoculation. Tomatoes were individually placed in zip-lock bags, stored in the greenhouse and sampled from 0 to 15 days post inoculation. At each sampling point, 3 tomatoes of each treatment were submerged in 70% alcohol for 30 s, 0.6% sodium hypochlorite for 20 s and finally rinsed with SDW twice. The pulp of the tomatoes was extracted and analyzed for *S. enterica* Typhimurium CFUs as described above.

### Growth of *S. enterica* Typhimurium at a range of pH levels

Experiments were conducted to determine the pH values at which *S. enterica* Typhimurium MAE110 and MAE119 could grow at room temperature. The experiments used 50 ml of liquid LB in 250 ml flasks as a base medium and were repeated twice on different dates. Hydrochloric acid was used to adjust the pH of the media to a range between 2.2 and 7 with a 0.4 unit interval as described previously [48]. Each strain was replicated in three flasks in each experiment. The inoculum of *S. enterica* Typhimurium was prepared as described above. Fifty µl suspension (10^4^ CFU/ml) was added into each flask. After 3-day incubation at room temperature, 0.5 ml of medium suspension was transferred from each flask to determine the CFU of *Salmonella*. The dilution series and plating were the same as described above.

### Plant dry weight measurements

Aboveground dry weights of tomato plants, after removal of fruits, were measured as described previously [49]. In brief, plants were removed from the soil and any loose soil was washed off; the plants were then blotted to remove any free surface moisture and dried in an oven at 37±2°C for 4 days. The dry weights were measured after the plants cooled in zip-lock bags.

### Statistical analysis

The number of colonies per plate was converted to CFU/ml or CFU/g (fresh weight) and log-transformed to obtain normal distributions for statistical analysis. The surface disinfection effect and the effect of bacterial rdar morphotype on the internal persistence of *S. enterica* Typhimurium in tomato leaves at the inoculation site was evaluated by fitting log-transformed data (separately for each replication) to the exponential decay model with asymptote: C_t_  = A+(M − A)e^−Rt^+E_t_ [50], in which C = *S. enterica* Typhimurium concentration (log (CFU/g)), A = asymptote (log (CFU/g)), M = initial bacterial concentration (log (CFU/g)), R = growth rate (day^−1^), t = time (day) and E = Error term. Estimated values of the parameters were subjected to multivariate analysis of variance (MANOVA). Similarly, log-transformed data of *Salmonella* concentration in tomato fruits through peduncle injection were fitted to the Gompertz equation: Y_t_ 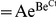
+E_t_, in which Y = *S. enterica* Typhimurium concentration (log (CFU/g)), A = upper asymptote (log (CFU/g)), B = growth displacement (dimensionless), C = growth rate (day^−1^), t = time (day) and E = Error term. Statistical analyses (ANOVA, MANOVA, nonlinear regressions, and t tests) were performed using SAS (SAS release 9.2, SAS Institute Inc., Cary, NC).

## Results

### Plant surface disinfection efficiency

On average, 6.60×10^4^±1.10×10^4^ *S. enterica* serovar Typhimurium CFU were recovered after the alcohol/hypochlorite washes of the inoculated leaves, whereas 1.36×10^8^±0.28×10^8^ CFU were obtained in the absence of the leaf disinfection treatments. Thus, the treatment reduced counts by about 2000 times (3.3 logs), and the surface disinfection efficiency was about 99.95% (±0.21%). The surface disinfection efficiencies for *S. enterica* Typhimurium strains MAE110 and MAE119 were not significantly different (P>0.05).

### Effect of surfactants on *S. enterica* Typhimurium colonization in tomato leaves

**Figure 1:**
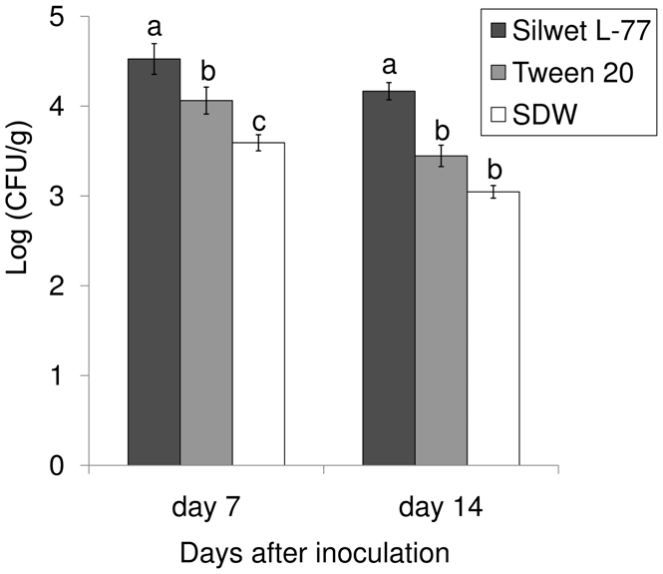
Survival of *Salmonella enterica* Typhimurium inside tomato leaves after *Salmonella* inoculation with or without surfactants. SDW: Sterile distilled water.

Seven and 14 days after inoculation, the population of *S. enterica* Typhimurium MAE110 in the tomato leaves inoculated with a suspension plus Silwet L-77 (4.53±0.09, 4.16±0.07 Log (CFU/g)) was significantly higher than that in leaves inoculated with a suspension with Tween 20™ (4.06±0.14, 3.45±0.11 Log (CFU/g)) or a suspension in SDW (3.59±0.17, 3.04±0.10 Log (CFU/g), Fig. 1). Thus, the application of Silwet L-77 to leaves may enhance the initial internalization or survival of *Salmonella* in tomato leaves.

### Surface and internal colonization of tomato leaves by *S. enterica* Typhimurium

**Figure 2:**
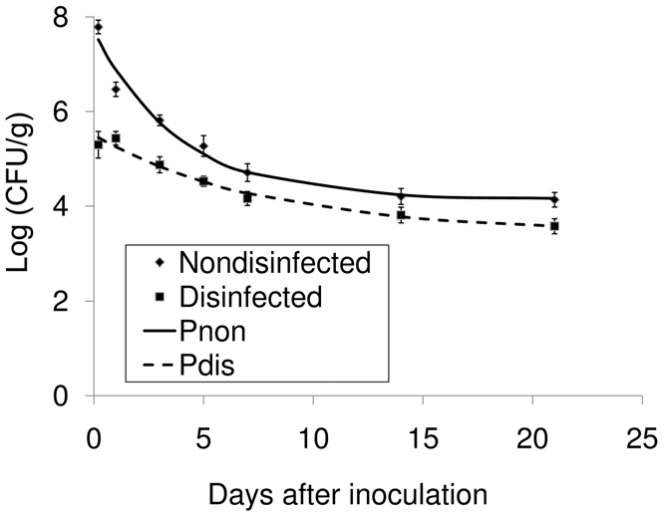
Survival of *Salmonella enterica* Typhimurium on/in tomato leaves with/without surface disinfection. Pdis and Pnon are the predicted regression curves based on an exponential decay model with asymptote for the survival of *Salmonella* with and without surface disinfection, respectively.

Three hours after inoculation, the *Salmonella* concentration on non-disinfected leaves was about 10^8^ CFU/g, which was about 3 logs higher than the concentration in disinfected leaves (∼10^5^ CFU/g) (Fig 2). Based on the disinfection efficiency described above, most of the bacteria (99.9%) were attached to the leaf surface at that time. After 1 day, the *Salmonella* concentration in disinfected leaves remained the same while the concentration of non-disinfected leaves significantly decreased. Additionally, the decrease rate of the *Salmonella* populations on non-disinfected samples was about two times as high as that in disinfected samples (Table 1). These results suggest that *Salmonella* survived better after internalization when compared to surface colonization.

**Table 1:** Statistical analysis of parameter estimates for the exponential decline of *Salmonella enterica* Typhimurium concentrations on/in tomato leaves over a 21 day period.

Experiment	Treatment	M 1 (log (CFU/g))	A 2 (log (CFU/g))	R 3 (day^−1^)
Surface disinfection (MAE110+119)	Non-disinfected	7.7036±0.1494 4, a	4.1093±0.4946^ a^	0.2676±0.0488^ a^
	Disinfected	5.5175±0.0743^ b^	3.4049±0.4036^ b^	0.1315±0.0580^ b^
Internal colonization	MAE110	5.4844±0.0978^ a^	3.7431±0.1690^ a^	0.0897±0.0421^ a^
	MAE119	5.5506±0.0203^ a^	3.0668±0.2160^ b^	0.1732±0.0564^ b^

1 Initial bacterial concentration;

2 Asymptote;

3 Growth rate;

4 Letters indicate significant differences (P = 0.05) between treatments within each of the experiments.

Two weeks post inoculation, the *Salmonella* concentration in disinfected leaves decreased to about 10^4^ CFU/g, which was about 0.5 to 1 log less than that of the non-disinfected leaves. The population on the surface was at least 2 times higher, and over 65% of *Salmonella* existed on the surface.

No *Salmonella* was detected in the control plants.

### Effect of bacterial rdar morphotype on the persistence of *S. enterica* Typhimurium inside tomato leaf tissues

**Figure 3:**
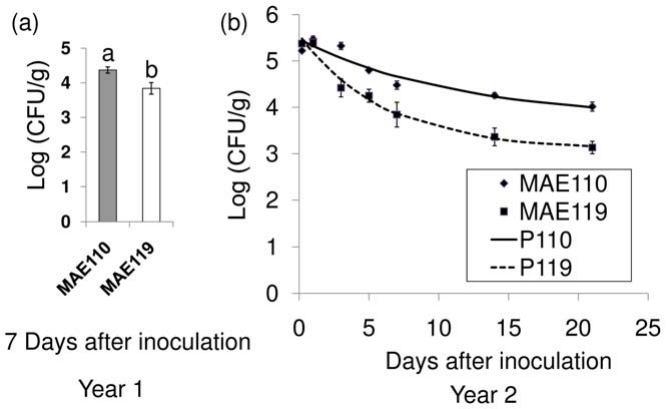
Population of *Salmonella enterica* Typhimurium strains MAE110 and MAE119 in tomato leaves after surface disinfection. Population of *Salmonella* strains MAE110 and MAE119 in inoculated tomato leaves 7 days after inoculation in year 1 (a); Survival trends of *Salmonella* strains MAE110 and MAE119 in inoculated tomato leaves in year 2 (b). P110 and P119 are the predicted regression curves based on the exponential decay model with asymptote for the survival of *Salmonella* strains MAE110 and MAE119.

In year 1, levels of *S. enterica* Typhimurium strain MAE110 (4.37±0.09 Log (CFU/g)) were significantly higher than those of strain MAE119 (3.84±0.17 Log (CFU/g)) at 7 days post inoculation (Fig 3a). In year 2, leaves were sampled several times between day 1 and day 21 to confirm the result obtained in the first year (Fig. 3b). The populations of both *Salmonella* strains in surface disinfected leaves decreased during the first 2 weeks after inoculation but remained unchanged in week 3. The exponential decay model used to describe survival of *Salmonella* in each sample had a mean square error of 0.257 and a coefficient of variation (R^2^) of 0.974. With respect to estimates of R (rate), A (asymptote) and M (initial bacterial concentration), the two strains were significantly different, with an overall Wilk's Lambda significance value of 0.0228 (Table 1). The R and A values of MAE110 were significantly higher than those of MAE119 (P<0.05), while M was not significantly different. Thus, *S. enterica* Typhimurium strain MAE110 with rdar morphotype persisted longer inside tomato leaves than the saw morphotype strain MAE119.

### Internalization and movement of *S. enterica* Typhimurium in tomato plants

**Figure 4:**
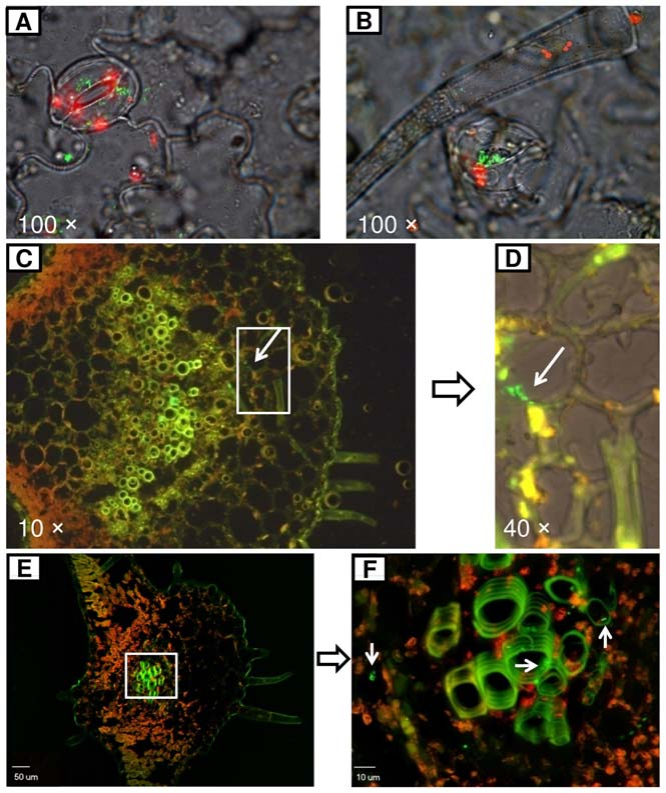
Microscopy of inoculated tomato leaf tissue sections colonized by *Salmonella enterica* Typhimurium. Fluorescence microscopic images of GFP-tagged *Salmonella* (green) showing both diffuse and stomata-associated attachment on inoculated leaves. Red fluorescence is the autofluorescence of plant chloroplasts (A and B). Endophytically present *Salmonella* was observed in the mid-rib vein of inoculated tomato leaves (C and D) and inside the vascular system (E and F). Image F as merged image under GFP and TRITC filters (Figure S1) was obtained by projecting 15 Z section overlaid fluorescence images of different layers (Figure S2) with 1 um interval into one combined image. Fluorescence and confocal microscopic images were labeled with magnification and scale bars, respectively.

**Figure 5:**
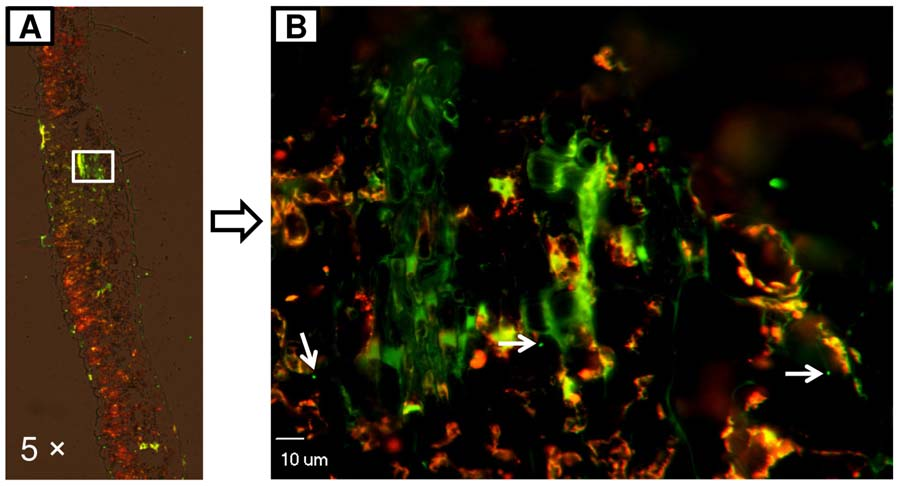
Microscopy of non-inoculated tomato leaf tissue sections colonized by *Salmonella enterica* Typhimurium. *Salmonella* was observed inside the non-inoculated leaves close to the veins. Image B as merged image under GFP and TRITC filters (Figure S3) was obtained by projecting 15 Z section overlaid fluorescence images of different layers (Figure S4) with 1 um interval into one combined image. Fluorescence and confocal microscopic images were labeled with magnification and scale bars, respectively.

**Figure 6:**
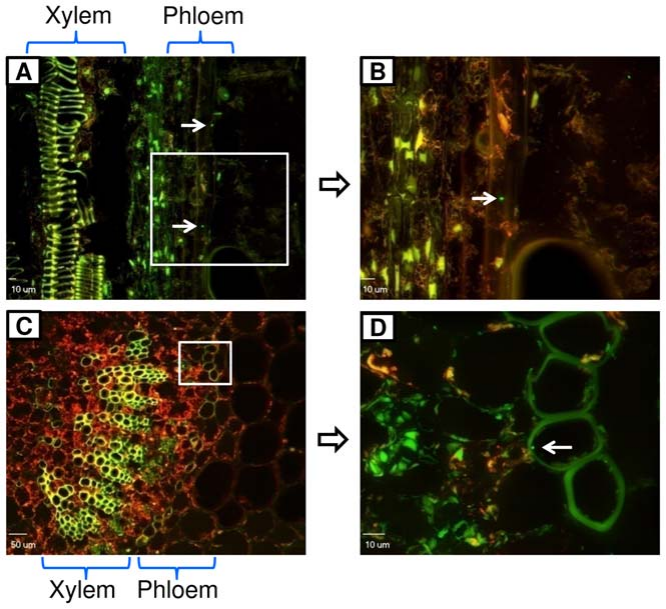
Confocal microscopy of non-inoculated tomato stem tissue sections colonized by *Salmonella enterica* Typhimurium. *Salmonella* was located in the phloem of non-inoculated stems in vertical plant tissue cross sections (A and B) and horizontal sections (C and D). Images B and D as merged images under GFP and TRITC filters (Figure S5, S7) were obtained by projecting 15 Z section overlaid fluorescence images of different layers (Figure S6, S8) with 1 um interval into one combined image.

In year 2, *Salmonella* was detected in adjacent non-inoculated leaves of eight tomato plants at 5 days post first inoculation (five plants inoculated with strain MAE110 and three with strain MAE119) (Table 2). To confirm the internalization and movement of *S. enterica* Typhimurium in tomato plants, plant tissues were sampled from these 8 tomato plants after the second inoculation. *Salmonella* cells were observed on the leaf surface, frequently associated with the trichomes and sometimes harbored by stomata at a rate of about 2–3% of the stomata (Fig. 4 A and B). One day after inoculation, *Salmonella* cells had ingressed into tomato leaves, moved into midrib veins of leaves (Fig. 4 C and D) and sometimes entered the vascular system, in particular the xylem (Fig. 4 E and F). As expected, *Salmonella* cells were also found inside non-inoculated leaflets adjacent to the inoculated leaflets on the eight plants where non-inoculated adjacent leaflets had tested positive for *Salmonella* (Fig. 5, Table 1). In addition, *Salmonella* was detected in the adjacent non-inoculated stems, including inside the phloem (Fig. 6). The frequency of *Salmonella* detected in non-inoculated adjacent leaves and stems was not very high (leaf cross section slides: 11 positive out of ∼960; stem slides: 5 positive out of ∼240). The presence of *Salmonella* cells as projected images of several Z section-overlaid fluorescence images from different layers (Fig. 4 F, Fig. 5 B, Fig. 6 B and D) indicated that the bacterial cells were located inside the plant tissues (Figures S2, S4, S6, S8), and that the presence of GFP fluorescent cells was not caused by contamination during manipulation. In addition, the observation of the *Salmonella* cells in the images obtained under a GFP filter (green fluorescence), and absent under a TRITC filter (red auto-fluorescence of chloroplasts and vascular tissues) confirmed that they were GFP labeled bacterial cells instead of plant tissues with auto-fluorescence (Figures S1, S3, S5, S7). All these microscopic results supported the internal movement of *S. enterica* Typhimurium in tomato plants. *Salmonella* cells were not detected in samples of control plants.

**Table 2:** *Salmonella enterica* Typhimurium contamination in tomato plants.

Year	Treatment	No. of internally contaminated plants/ total plants^1^	No. of plants with contaminated fruit/ total plants	No. of contaminated fruits/total fruits
1	MAE110	-	0/42	0/270
	MAE119	-	1/42	9/270^2^
	SDW	-	0/42	0/270
2	MAE110	5/42	2/42	7/250^3^
	MAE119	3/42	0/42	0/250
	SDW	0/42	0/42	0/250

1 Plants with internally contaminated non-inoculated leaflets adjacent to inoculated leaflets.

2 9/9 fruits on one plant;

3 5/6 fruits on one plant; 2/7 fruits on the other plant.

### Colonization of fruit pulp by *S. enterica* Typhimurium

**Figure 7:**
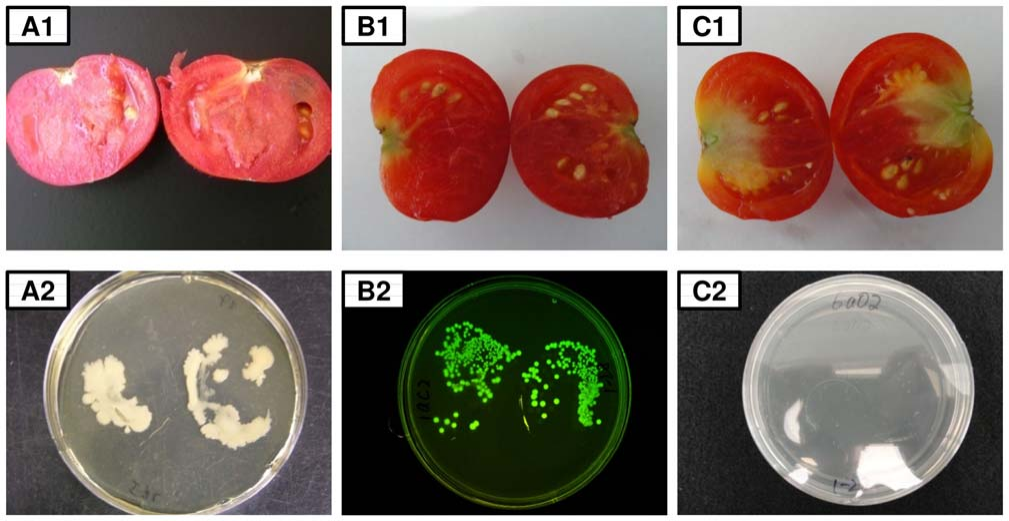
Tomato fruit contamination of *Salmonella enterica* Typhimurium. A1 and B1 are the cut fruits from *Salmonella* strains MAE110 and MAE119 contaminated plants, respectively. A2 and B2 present the *Salmonella* colonies recovered from corresponding fruits shown in A1 and B1 on LB plates with kanamycin; GFP labeled *Salmonella* colonies showing green fluorescence under UV lamp (B2). C1 and C2 are controls.

In the first year experiment, a total of 810 tomato fruits collected from the 126 (84 inoculated with *Salmonella* and 42 with SDW) tomato plants were tested for the presence of *Salmonella* on the surface of fruit or in tomato pulp. *S. enterica* Typhimurium was not detected in the wash water after enrichment, indicating that the fruit were not externally contaminated. One of the 42 MAE119-inoculated plants was systemically infected by *S. enterica* Typhimurium (Table 1). All nine fruits collected from that plant were internally colonized at high concentrations while no symptoms were observed (Fig. 7 A1 and A2). In year 2, a total of 750 tomato fruits were tested for the presence of *Salmonella* on the surface of fruits and in tomato pulp. Again, *S. enterica* Typhimurium was not detected in the wash water after enrichment. Two of seven harvested tomatoes of one plant and five of six harvested tomatoes from another plant were found *Salmonella*-positive, and both of these two plants were inoculated with strain MAE110. Both of these plants also tested positive for *Salmonella* in adjacent non-inoculated leaves (Table 1). Six of these contaminated fruits from the two *Salmonella*-positive plants were located at lower positions on the plants, closer than 5 cm from the inoculated leaves. Only one colonized fruit was collected from the top of one systemically infected tomato plant suggesting that *Salmonella* may not have moved very far up in the plants. The average concentration of *S. enterica* Typhimurium in the colonized tomato fruits was 6.3×10^5^±1.9×10^5^ CFU/g. The lack of visible symptoms and the distributions of the bacterial cells in the pulp are shown in Fig. 7, where A1 and B1 are the cut fruits from *Salmonella* strains MAE119 and MAE110 contaminated plants, respectively. A2 and B2 show the *Salmonella* colonies recovered from the corresponding fruits shown in A1 and B1 on LB plates with 50 µg/ml kanamycin, the GFP-labeled *Salmonella* colonies showed green fluorescence under a UV lamp (B2). Fig. 7 C1 and C2 show controls.

**Figure 8:**
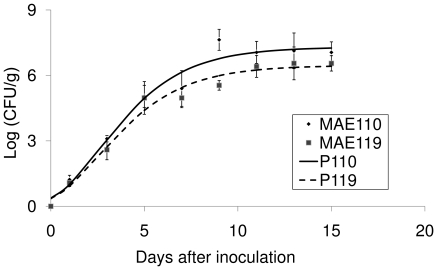
Growth of *Salmonella enterica* Typhimurium strains in tomato fruits after injection through peduncles. P110 and P119 are the predicted regression curves based on the Gompertz equation for the growth of *Salmonella* strains MAE110 and MAE119.

**Figure 9:**
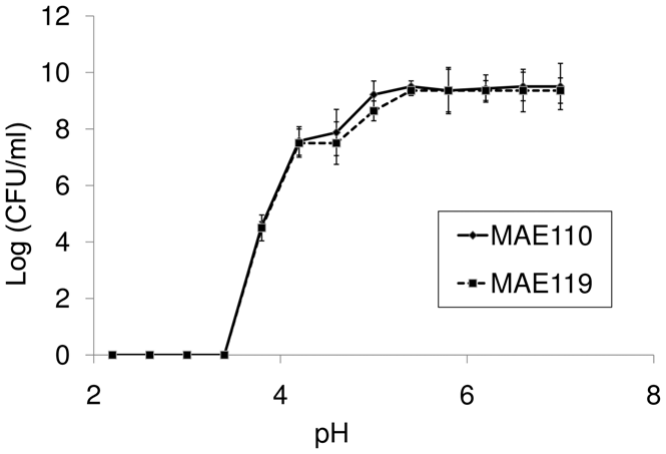
Growth of *Salmonella enterica* Typhimurium strains MAE110 and MAE119 at low pH levels. The concentrations of strains MAE110 and MAE119 were determined 3 days after inoculation.

*S. enterica* Typhimurium reached the interior of the fruit that were inoculated in the peduncle and multiplied inside the pulp to concentrations of about 10^7^ CFU/g pulp (fresh weight). The Gompertz model for growth of *Salmonella* in MAE110 and MAE119 of inoculated fruit had mean square errors of 0.2582 and 0.1653 and R^2^ values of 0.994 and 0.995, respectively. Estimates of A (asymptote), B (growth displacement) and C (growth rate for the two strains were not significantly different with an overall Wilk's Lambda significance value of 0.1383 (Fig. 8, Table 3). The population of *Salmonella* reached over 10^6^ CFU/g in LB media when the pH was above 4 (Fig. 9). There was no significant difference between the log(CFU/ml)s of two *Salmonella* strains at each pH condition. These results support the ability of *Salmonella* to multiply inside harvested tomato fruits, no matter where it was located inside the fruits.

**Table 3:** Statistical analysis of parameter values for a Gompertz growth curve of *Salmonella enterica* Typhimurium in tomato fruits after peduncle injection.

*Salmonella* strains	A 1 (log (CFU/g))	B 2 (dimensionless)	C 3 (day^−1^)
MAE110	7.3047±0.6040 4, a	−2.9808±0.0548^ a^	−0.4067±0.0132^ a^
MAE119	6.4617±0.3953^ a^	−2.9432±0.0916^ b^	−0.4075±0.0334^ b^

1 Asymptote;

2 Shoulder;

3 Growth rate;

4 Letters indicate significant differences (P = 0.05) between treatments.

### Effect of *S. enterica* Typhimurium inoculation on aboveground dry weight of tomato plants

**Figure 10:**
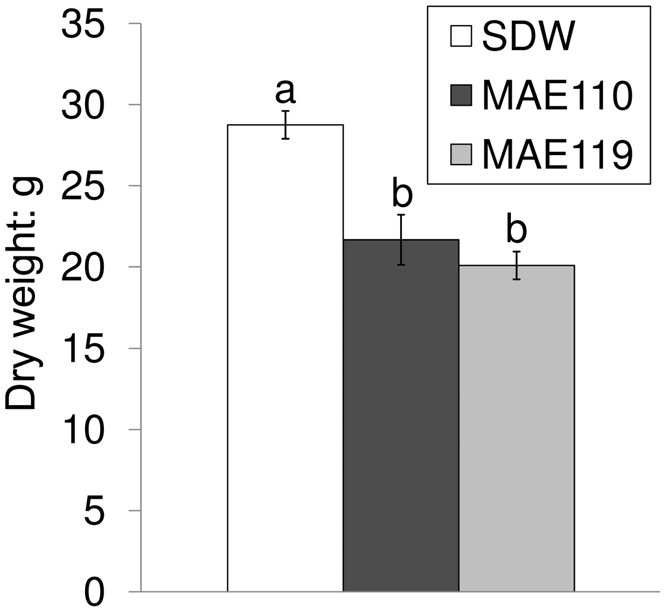
Dry weights of aboveground parts of tomato plants after treatment with *Salmonella enterica* **Typhimurium.** SDW: Sterile distilled water.

Aboveground parts of tomato plants were collected at the end of the second experiment (5 months growth) and dried for weight measurements (Fig. 10). Compared to the plants treated with SDW containing 0.025% (v/v) Silwet L-77, the aboveground dry weights of the plants inoculated with *Salmonella* were significantly decreased, indicating that *Salmonella* inoculation could reduce the aboveground plant biomass. During the experiment, the inoculated tomato leaves turned yellow, wilted and finally dropped. While the leaves inoculated with SDW with 0.025% Silwet L-77 remained healthy. Thus, the reduction in biomass may be partially due to the drop of inoculated leaves.

## Discussion

The main results obtained from this research were that *S. enterica* Typhimurium entered tomato plants via the leaves (possibly through stomates) and moved through petioles and stems into non-inoculated leaves and fruits, although the rate of internal fruit contamination was low. The rdar mophotype of *S. enterica* Typhimurium enhanced the ingress and internal persistence in tomato leaves at the inoculated sites. This is the first time to confirm that *S. enterica* can be transported inside tomato plants to contaminate fruits internally, possibly by moving through phloem, the main means of transportation of liquid and sugars into the fruit [51]. Previous studies demonstrated that the inoculation of flowers and stems with *S. enterica* can result in the contamination of tomato fruits [52], and that inoculation of leaves can result in surface contamination of tomato fruits [41]. However, the internal movement of *Salmonella* from leaves into tomato fruits has not been reported.

To investigate the presence of *S. enterica* Typhimurium inside plant tissues, the *Salmonella* cells on the plant surface must be removed efficiently without killing the bacteria inside the plant. For this purpose, the efficiency of 70% ethanol and 0.6% sodium hypochlorite was evaluated for surface disinfection of the leafy parts of plants that were dip-inoculated with *S. enterica* Typhimurium. The decrease of the *Salmonella* population after the disinfection treatment was about 3.3 logs which is higher than the 2.7 logs reduction shown by Klerks [42], mainly due to the longer disinfection time and additional treatment with sodium hypochlorite in this experiment.

The *Salmonella* rdar mophotype is important for the attachment to plant surfaces [30], [53] and the persistence in environments outside of animal hosts [33], [37], but it may not be critical to the persistence within tomato fruits [54]. In this study, the results indicated that *Salmonella* stain MAE110, permanently containing the rdar morphotype, survived better in inoculated tomato leaves compared to the rdar deficient mutant strain MAE119. However, the contamination rate in adjacent leaves and fruits (Table 2) was not significantly higher for strain MAE110. A possible explanation may be that characteristics associated with the rdar morphotype protected the bacteria from stress on the surface and just below the surface of inoculated leaves, but these character traits were less important once the cells had completely entered the plants and were sheltered from external stress factors. Another explanation may be that the *Salmonella* rdar mophotype may have a different function in tomato leaves than in fruits. Further molecular biological studies should be conducted to investigate the mechanism how the rdar mophotype affects the survival of *Salmonella* inside plant leaves, stems and fruits.

During our microscopic observations, we noticed that *S. enterica* Typhimurium was frequently observed at the base of leaf trichomes (data not shown), similar to a previous report on the distribution of a mixture of strains of *S. enterica* (not including serovar Typhimurium) on tomato leaf surfaces [41]. In that report, *S. enterica* cells were not found in stomates. We observed that *S. enterica* Typhimurium cells were located in stomates and that inoculation did not result in stomatal closure, similar to the colonization of *S. enterica* Typhimurium on iceberg lettuce [55]. Thus, *Salmonella* cells could have entered through these “open gates” (Fig. 4 A and B). In our study, 2–3% of the stomata of inoculated leaves contained *S. enterica* Typhimurium cells. So, besides wounds, stomata may be an important pathway for *Salmonella* ingress into tomato leaves.

Although *Salmonella* cells were observed in the vascular system of inoculated leaves, they were not found in the xylem vessels of non-inoculated plant tissues. Yet, they were observed in the phloem of non-inoculated tissues. These results suggest that phloem is more conducive for presence of *Salmonella* when compared to xylem, probably due to the high levels of sugars and nutrients in the phloem. When lettuce or *Medicago truncatula* plants were grown in contaminated manure-amended soil or were inoculated on agar media, *S. enterica* infected the plants as a plant pathogen, invoking host defense responses [42], [56]. Similar to the findings of Klerks *et al.* [42], inoculated leaves became chlorotic and the biomass of inoculated plants was reduced in our experiments. This indicates that *S. enterica* Typhimurium had some pathogenic effect on tomato plants. However, the rare occurrence of *Salmonella* cells in the phloem of inoculated plants indicated that *Salmonella* was an exogenous bacterium in tomato plants, mainly colonizing the apoplast of the tissues [57]. Nevertheless, it could enter the vascular system in inoculated leaves, survive and move in the sieve tissues of the phloem and thus result in internal contamination of tomato fruits, although at a low rate (5 in 240 microscopic slides from 8 *Salmonella* positive plants). Unlike plant pathogens, which could produce hemicellulase and pectinases to degrade plant cell walls, the mechanism how *Salmonella* cells enter and survive inside the phloem is still unclear. One possibility for the rare occurrence of *S. enterica* Typhimurium in the phloem is that the primary sugar transported by the phloem in tomatoes is sucrose which could not be digested by *Salmonella* [58]. Another hypothesis is that the high concentration of sugars and other nutrients in phloem provides a negative osmotic pressure to the bacteria and limits water absorbability. Further studies would need to be conducted to answer these questions.

To confirm the possibility of internal growth of *S. enterica* inside tomato fruits, young pink fruits (pH 4–4.5) in this experiment were harvested and injected with low concentrations of *S. enterica* Typhimurium through the peduncle, and the growth of *S. enterica* Typhimurium was tested *in vitro* at a range of pH levels. *S. enterica* Typhimurium entered the fruit through the peduncle and multiplied inside the pulp. Similar as reported previously [59], *Salmonella* could grow when the pH was above 4. Although it is not exactly known whether *Salmonella* was in the symplast or apoplast inside the fruit and the pH values of various tissues in tomato fruits differ, a low pH value of any tissue in the tomato fruits would not be a limitation for *Salmonella* multiplication. Further studies would be needed to investigate the exact location of *Salmonella* in contaminated tomato fruits.

Based on the fruit contamination rate (Table 2), internal contamination is a low chance event, even though we set up a worst case scenario. To maximize the possibility of internalization, we inoculated tomato leaves two or four times before fruit set with a suspension of *S. enterica* Typhimurium at a high concentration (10^9^ CFU/ml) including the surfactant Silwet L-77, which could facilitate entry of bacteria into plant leaves [18]. The contamination rates of adjacent non-inoculated leaves and fruits were 9.5% and 1.8%, respectively, and the chance to detect contaminated fruits after inoculation was less than 1.5%. Nevertheless, due to the very large numbers of tomatoes produced in the USA, about 4 million metric tons in North America in 2003 [60], this low probability event would have a chance to occur, especially in large tomato fields with a high plant density (about 2×10^4^ plants / ha). Because the probability of internal movement of *Salmonella* in tomato plants is low, a high concentration of inoculum was necessary to obtain positive results for this fundamental research to investigate if internal movement was at all possible. In environmental samples such as manure that can be used to amend soil, *Salmonella* can be present in levels up to 10^6^ CFU/g [61] and grow to levels above 10^9^ CFU/g if microbial competitors are not present [62]. However, these conditions and high inoculum levels of *Salmonella* would be hard to reach in natural environments. A probabilistic microbial risk model would need to be developed to assess the contamination probability in a practical tomato production chain [63].

Another important point of this study is that all tomato plants were grown in agricultural soils collected from farms with a long cropping history. Unlike commercial potting mix, which usually contains more nutrients for plant growth and has excellent drainage properties [64], the agricultural soil we used reflected the conditions of a regular field, possibly providing a higher chance for survival and ingress into the plant and internal contamination of the fruit by *Salmonella* [65], [66]. Moreover, natural soil may also provide the right conditions for seed contamination, as the seeds extracted from the contaminated fruits in these experiments were internally contaminated by *Salmonella* (Gu and van Bruggen, to be published). Further studies to see if *S. enterica* Typhimurium could be transmitted from these internally contaminated seeds to seedlings, plants and fruits in the second generation are currently underway.

Similar as reported for lettuce [42], the biomass of tomato plants was reduced after inoculation of *Salmonella*. Further studies are needed to assess the mechanisms of plant biomass reduction by *Salmonella* compared with other bacteria.

The practical implication of this work may be that application of surfactants, especially Silwet L-77, could enhance the entrance of bacterial pathogens into leaf tissues (this work and [18]), although internal movement of *Salmonella* in tomato plants was not enhanced by surfactants. Additional experiments would be needed to investigate if a reduction in the application of fungicides, insecticides and herbicides containing surfactants could lower the risk of contamination with *S. enterica*.

### Conclusion

This work resulted in two major findings, viz. that *S. enterica* Typhimurium can reach tomato fruit via internal translocation from leaves through stems and that phloem tissue is a potential conduit. The chance of internal movement is low, but once *Salmonella* cells reach a fruit they can multiply to high densities within that fruit. Additional findings were that the rdar morphotype and surfactants enhanced initial colonization of leaf tissues.

## Supporting Information

Figure S1 Images of the same inoculated leaf section as in Figure 4F taken with GFP, TRITC filters and their combination. White arrows point at the locations of *Salmonella* cells shown with the GFP filter, and absence with the TRITC filter.
(TIF)

Figure S2 Images of the same inoculated leaf section as in Figure 4F obtained from different layers of a Z section. White arrows point at the locations of *Salmonella* cells inside the plant tissues.
(TIF)

Figure S3 Images of the same inoculated leaf section as in Figure 5B taken with GFP, TRITC filters and their combination. White arrows point at the locations of *Salmonella* cells shown with the GFP filter, and absence with the TRITC filter.
(TIF)

Figure S4 Images of the same inoculated leaf section as in Figure 5B obtained from different layers of a Z section. White arrows point at the locations of *Salmonella* cells inside the plant tissues.
(TIF)

Figure S5 Images of the same inoculated leaf section as in Figure 6B taken with GFP, TRITC filters and their combination. White arrows point at the locations of *Salmonella* cells shown with the GFP filter, and absence with the TRITC filter.
(TIF)

Figure S6 Images of the same inoculated leaf section as in Figure 6B obtained from different layers of a Z section. White arrows point at the locations of *Salmonella* cells inside the plant tissues.
(TIF)

Figure S7 Images of the same inoculated leaf section as in Figure 6D taken with GFP, TRITC filters and their combination. White arrows point at the locations of *Salmonella* cells shown with the GFP filter, and absence with the TRITC filter.
(TIF)

Figure S8 Images of the same inoculated leaf section as in Figure 6D obtained from different layers of a Z section. White arrows point at the locations of *Salmonella* cells inside the plant tissues.
(TIF)
