# Supplementary figures and images for: Internal Colonization of Salmonella enterica Serovar Typhimurium in Tomato Plants
Source: PLoS One. 2011 Nov 9;6(11):e27340. doi: 10.1371/journal.pone.0027340 (PMC3212569; doi:10.1371/journal.pone.0027340)

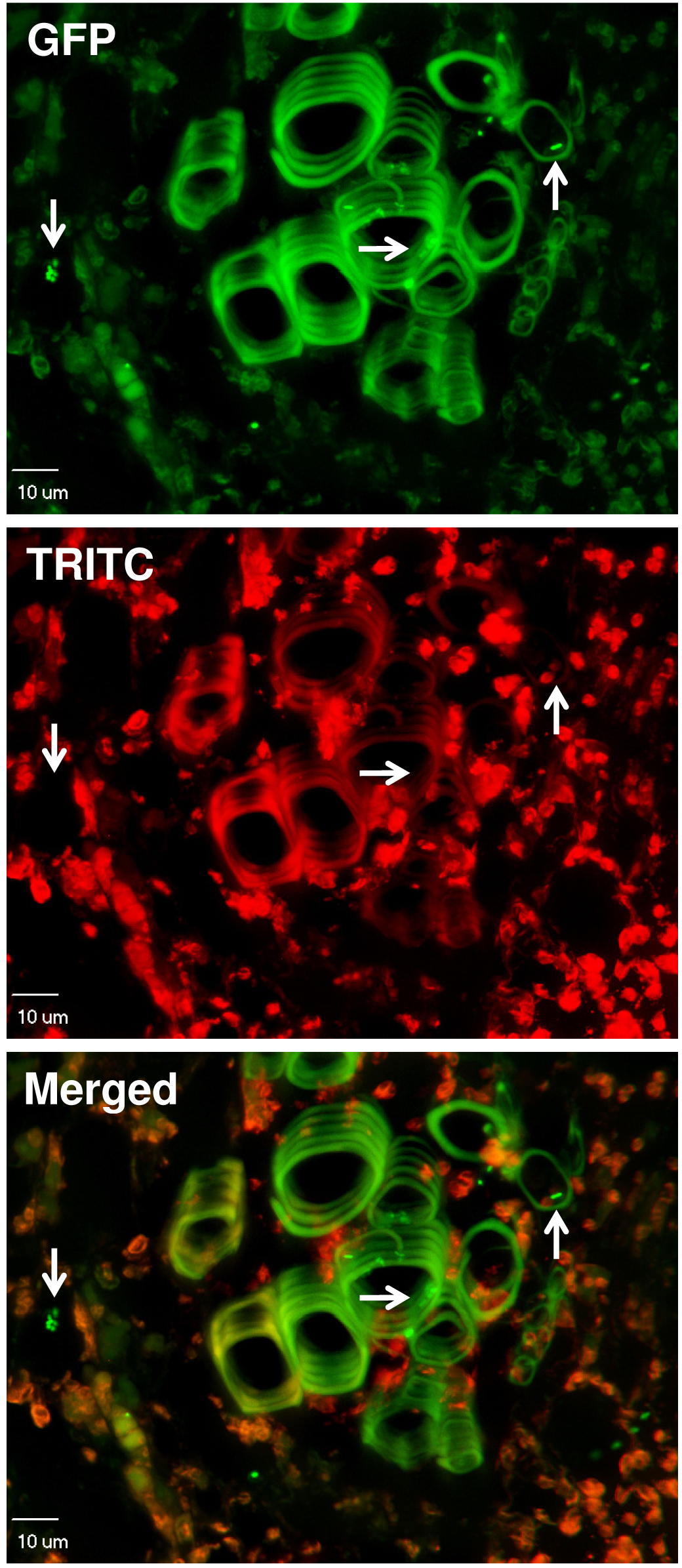

Supplement: Figure S1 — Images of the same inoculated leaf section as in Figure 4F taken with GFP, TRITC filters and their combination. White arrows point at the locations of Salmonella cells shown with the GFP filter, and absence with the TRITC filter. (TIF) [file pone.0027340.s001.tif]

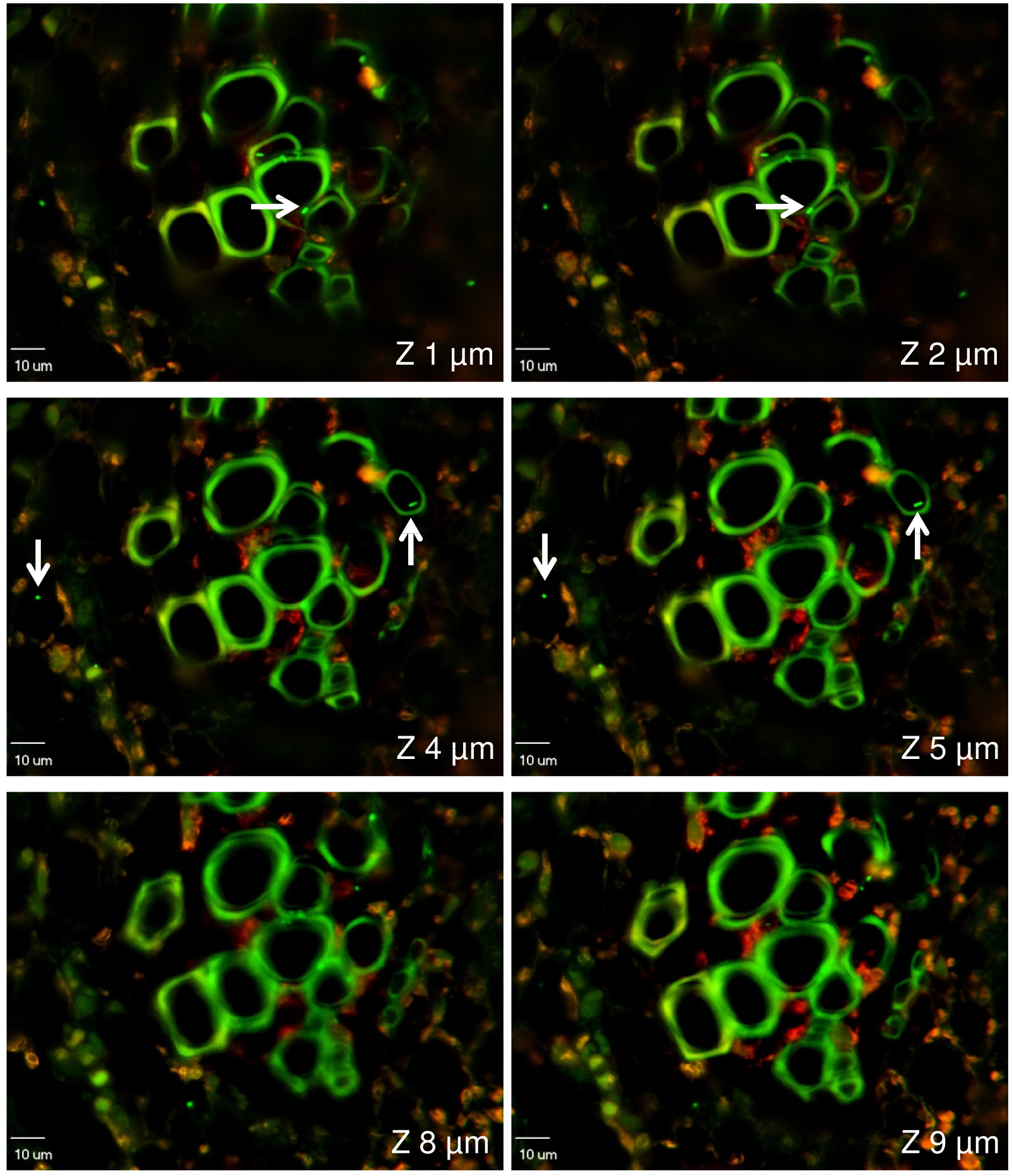

Supplement: Figure S2 — Images of the same inoculated leaf section as in Figure 4F obtained from different layers of a Z section. White arrows point at the locations of Salmonella cells inside the plant tissues. (TIF) [file pone.0027340.s002.tif]

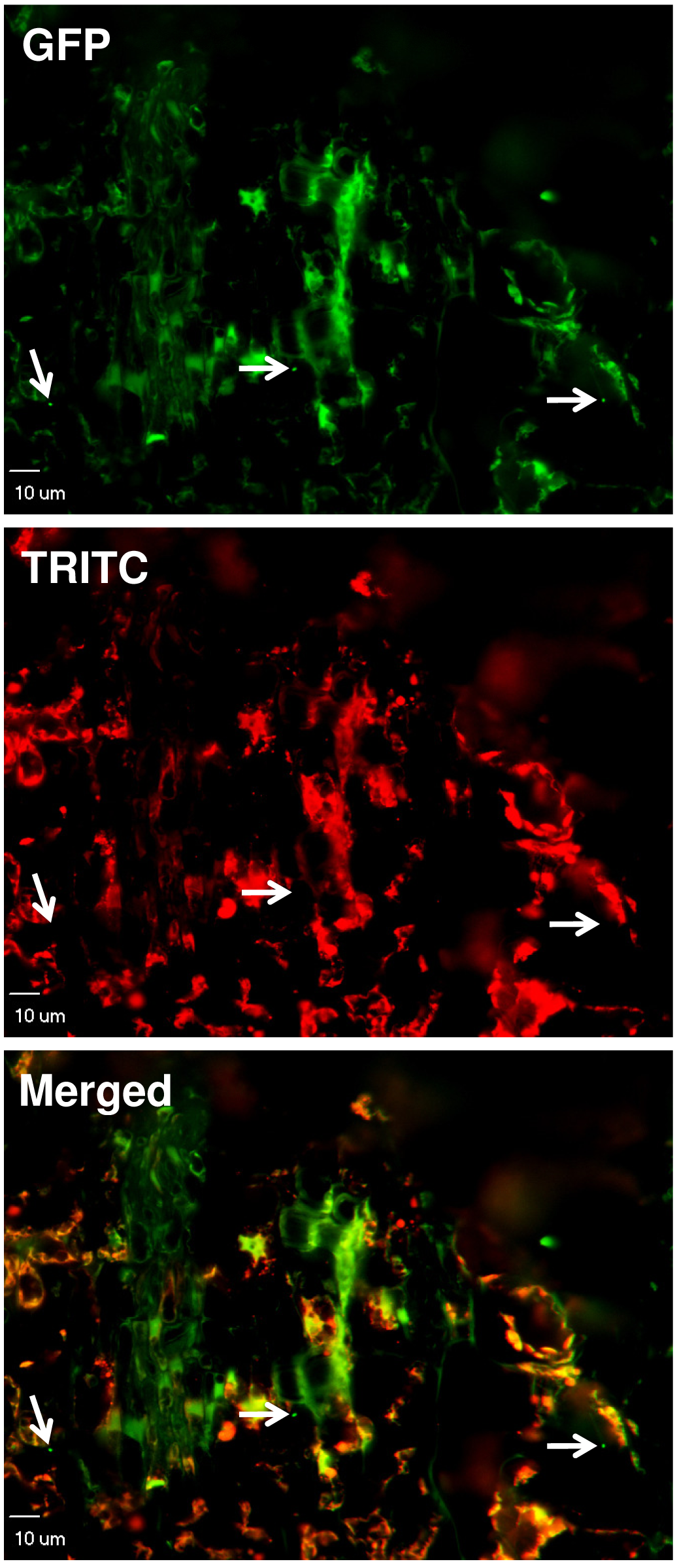

Supplement: Figure S3 — Images of the same inoculated leaf section as in Figure 5B taken with GFP, TRITC filters and their combination. White arrows point at the locations of Salmonella cells shown with the GFP filter, and absence with the TRITC filter. (TIF) [file pone.0027340.s003.tif]

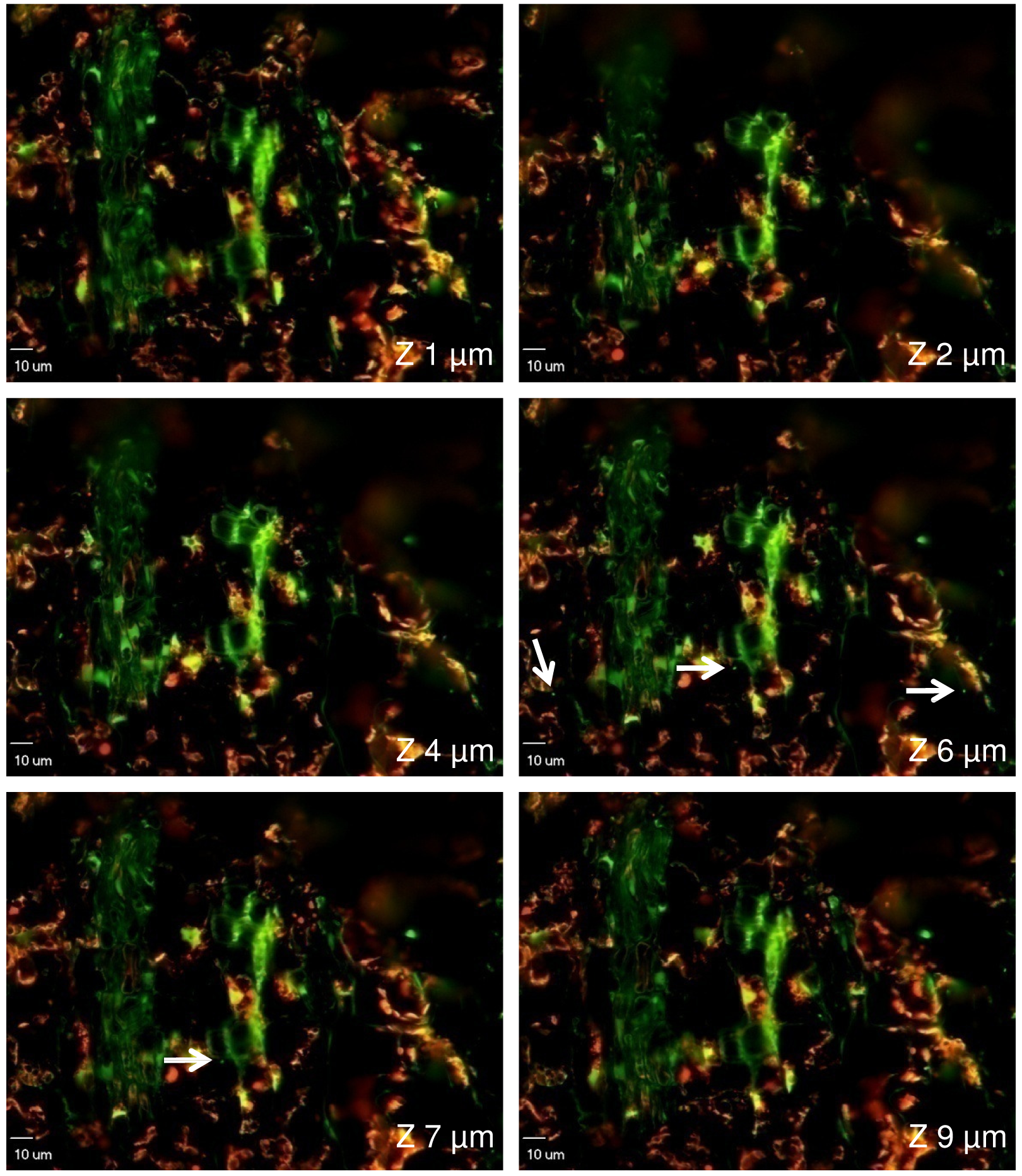

Supplement: Figure S4 — Images of the same inoculated leaf section as in Figure 5B obtained from different layers of a Z section. White arrows point at the locations of Salmonella cells inside the plant tissues. (TIF) [file pone.0027340.s004.tif]

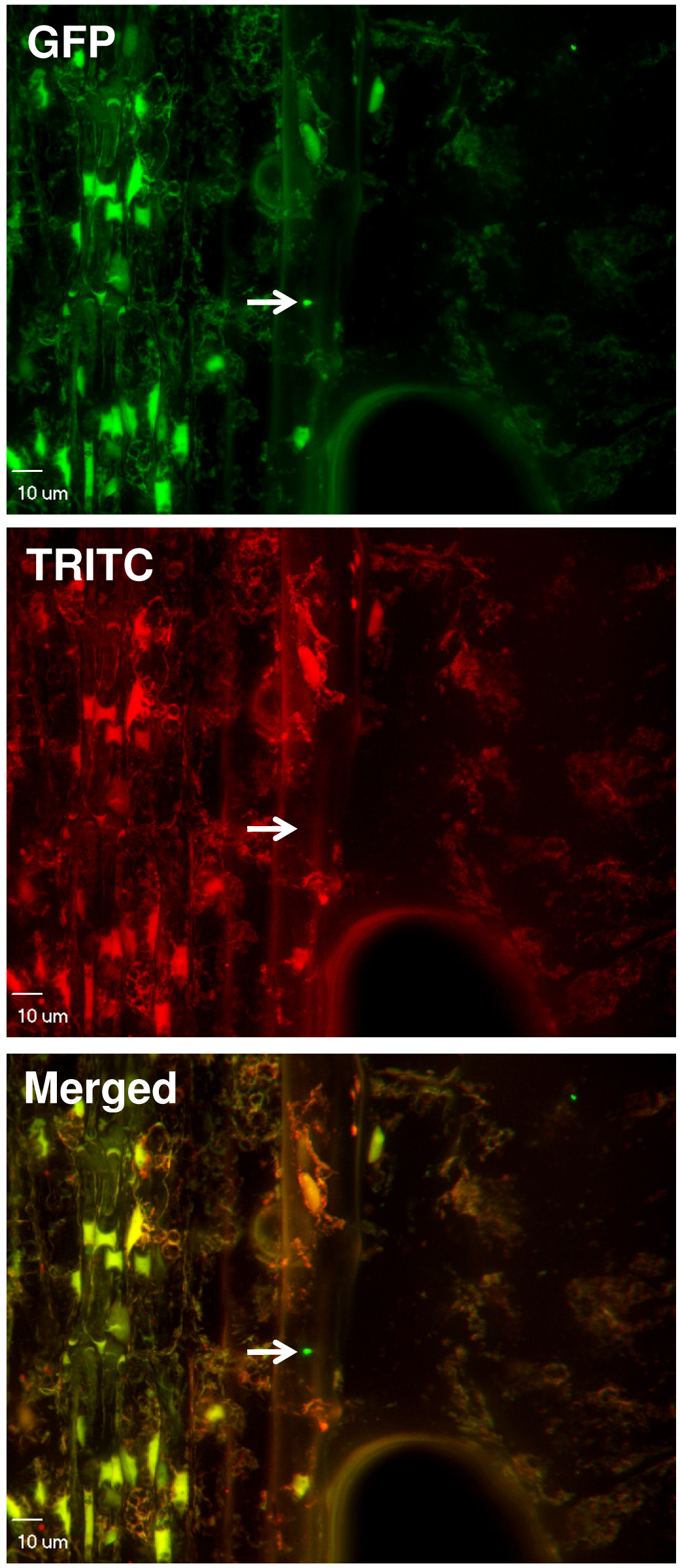

Supplement: Figure S5 — Images of the same inoculated leaf section as in Figure 6B taken with GFP, TRITC filters and their combination. White arrows point at the locations of Salmonella cells shown with the GFP filter, and absence with the TRITC filter. (TIF) [file pone.0027340.s005.tif]

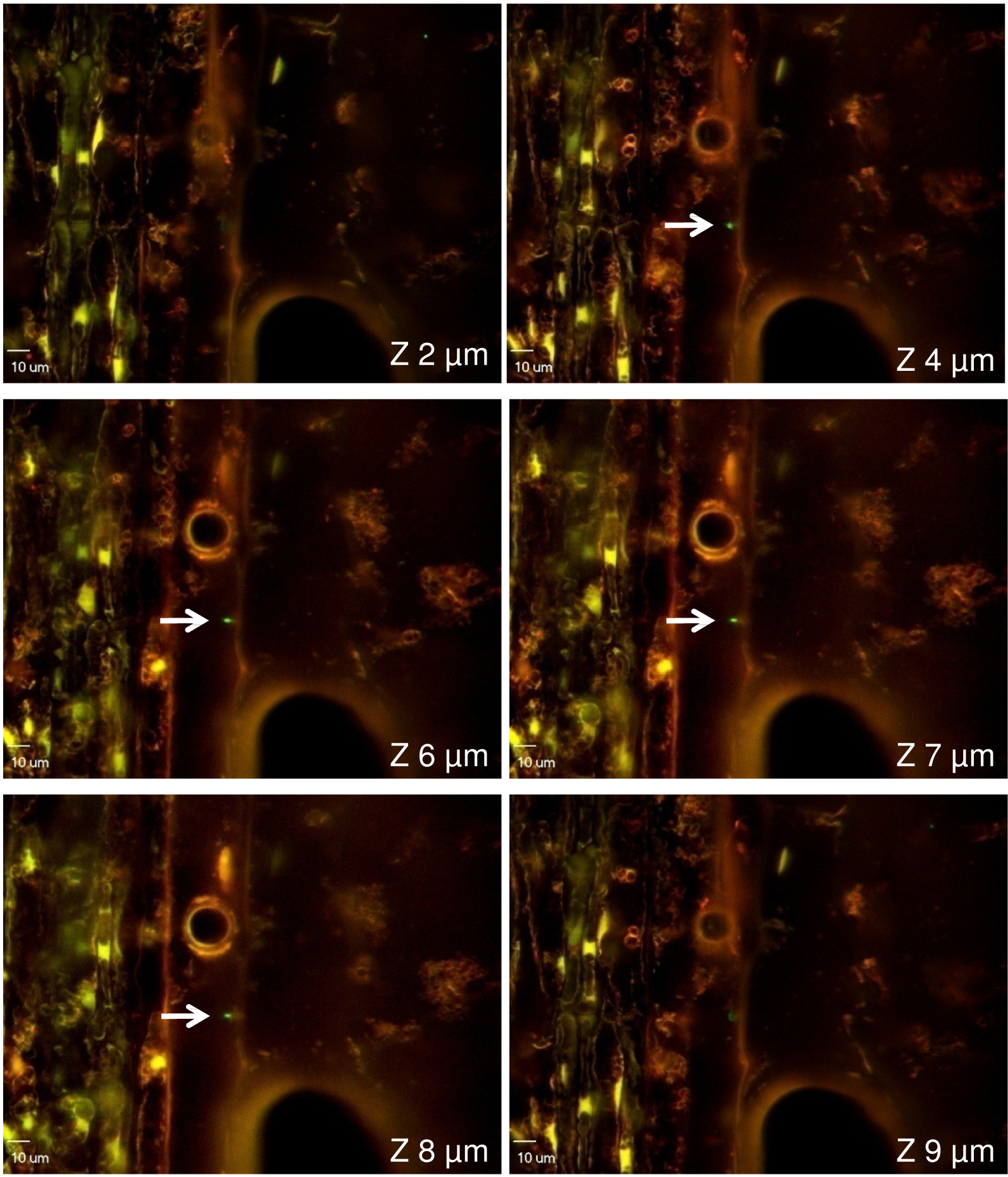

Supplement: Figure S6 — Images of the same inoculated leaf section as in Figure 6B obtained from different layers of a Z section. White arrows point at the locations of Salmonella cells inside the plant tissues. (TIF) [file pone.0027340.s006.tif]

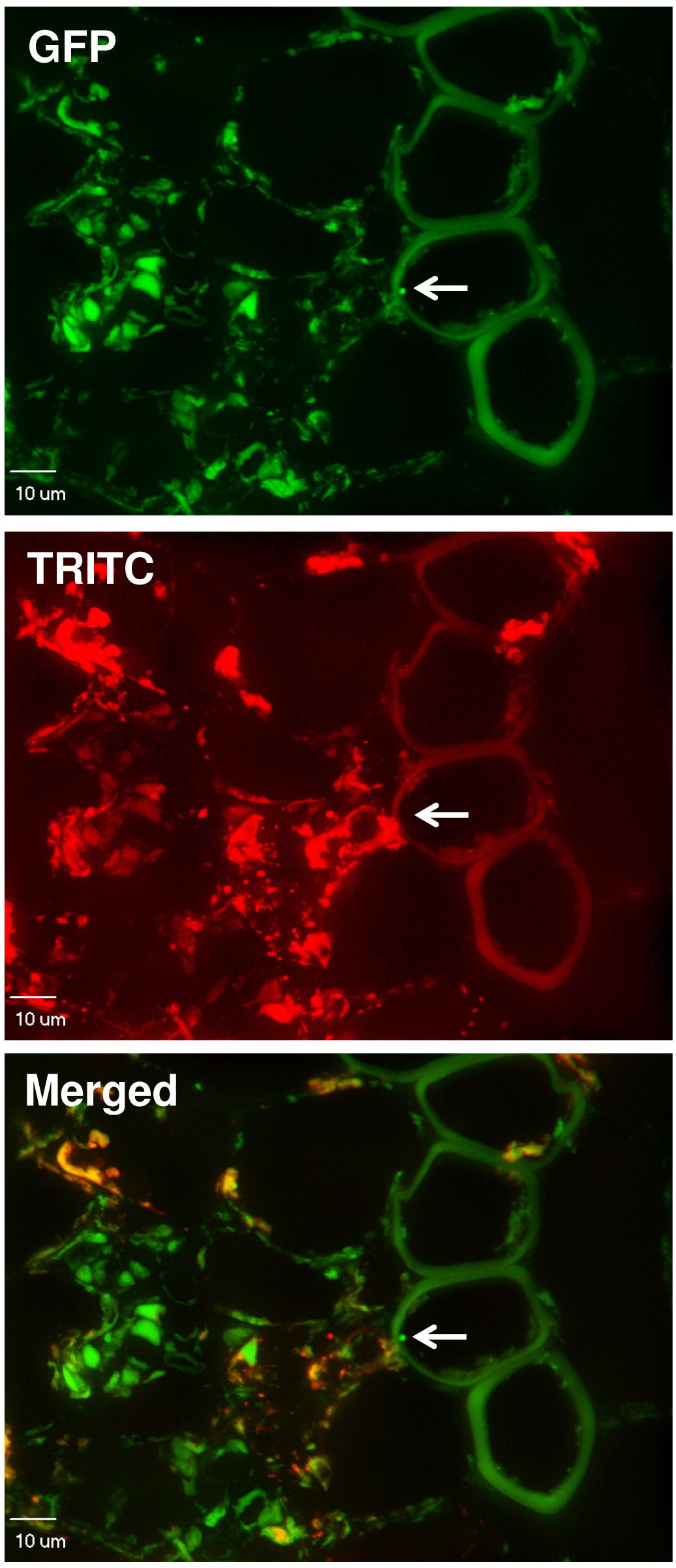

Supplement: Figure S7 — Images of the same inoculated leaf section as in Figure 6D taken with GFP, TRITC filters and their combination. White arrows point at the locations of Salmonella cells shown with the GFP filter, and absence with the TRITC filter. (TIF) [file pone.0027340.s007.tif]

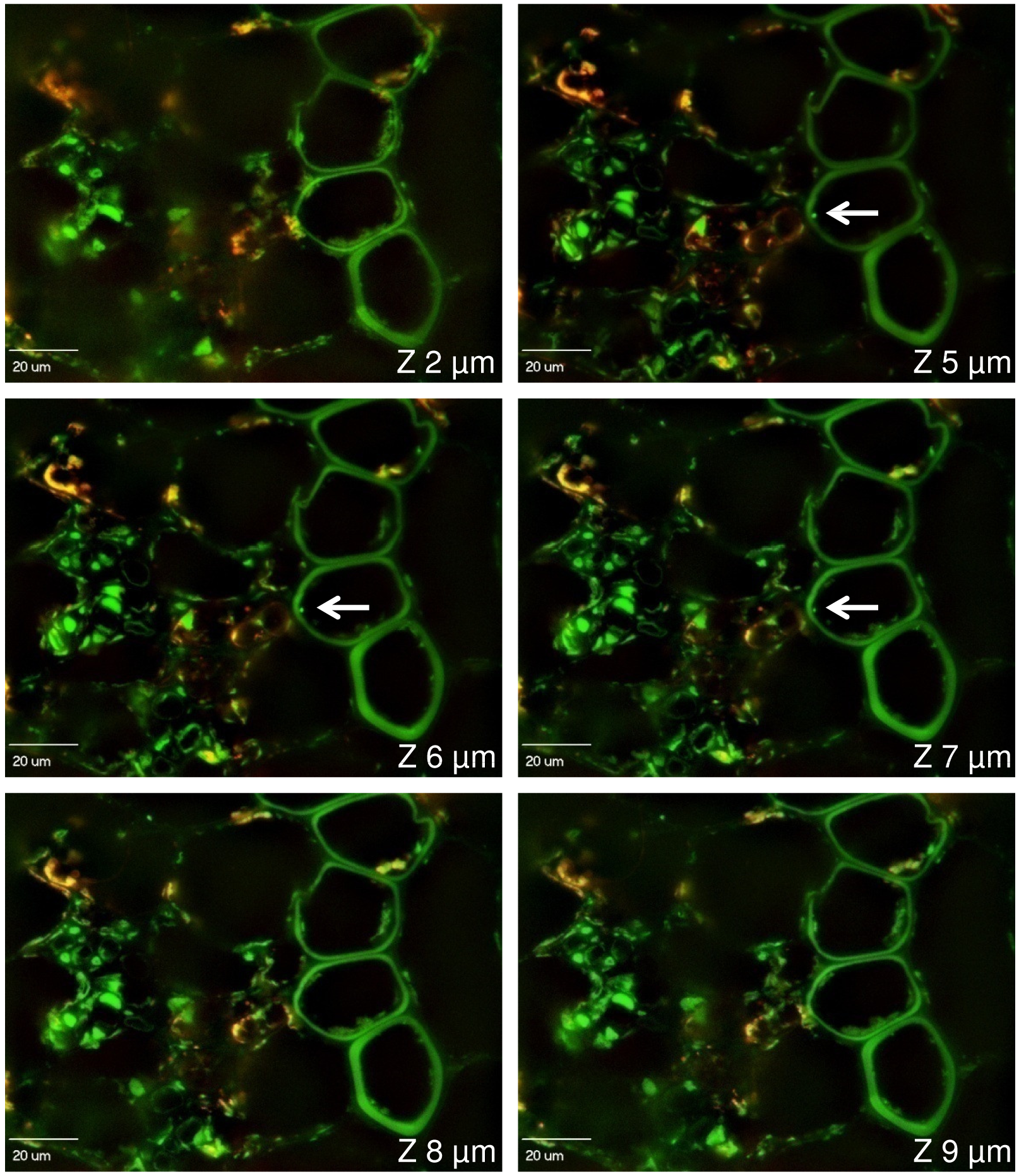

Supplement: Figure S8 — Images of the same inoculated leaf section as in Figure 6D obtained from different layers of a Z section. White arrows point at the locations of Salmonella cells inside the plant tissues. (TIF) [file pone.0027340.s008.tif]
